# Hyperlipidemia and mortality associated with diabetes mellitus co-existence in Chinese peritoneal dialysis patients

**DOI:** 10.1186/s12944-020-01405-5

**Published:** 2020-11-07

**Authors:** Xin Wei, Yueqiang Wen, Qian Zhou, Xiaoran Feng, Fen Fen Peng, Niansong Wang, Xiaoyang Wang, Xianfeng Wu

**Affiliations:** 1 Department of Nephrology, the First Affiliated Hospital of Nanchang University, Nanchang, China; 2 Department of Nephrology, the Second Affiliated Hospital of Guangzhou Medical University, Guangzhou, China; 3 Department of Medical Statistics, Clinical Trials Unit, The First Affiliated Hospital, Sun Yat-sen University, Guangzhou, China; 4 Department of Nephrology, Jiujiang No. 1 People’s Hospital, Jiujiang, China; 5 Department of Nephrology, Zhujiang Hospital of Southern Medical University, Guangzhou, China; 6 Department of Nephrology, Affiliated Sixth People’s Hospital, Shanghai Jiao Tong University, No.600, Yi Shan Road, Shanghai, China; 7 Department of Nephrology, The First Affiliated Hospital of Zhengzhou University, Zhengzhou, China

**Keywords:** Diabetes mellitus, Hyperlipidemia, Mortality, Peritoneal dialysis, Prognosis, Follow-up, Long-term survival

## Abstract

**Background:**

To evaluate associations between diabetes mellitus (DM) coexisting with hyperlipidemia and mortality in peritoneal dialysis (PD) patients.

**Methods:**

This was a retrospective cohort study with 2939 incident PD patients in China from January 2005 to December 2018. Associations between the DM coexisting with hyperlipidemia and mortality were evaluated using the Cox regression.

**Results:**

Of 2939 patients, with a median age of 50.0 years, 519 (17.7%) died during the median of 35.1 months. DM coexisting with hyperlipidemia, DM, and hyperlipidemia were associated with 1.93 (95% CI 1.45 to 2.56), 1.86 (95% CI 1.49 to 2.32), and 0.90 (95% CI 0.66 to 1.24)-time higher risk of all-cause mortality, compared with without DM and hyperlipidemia, respectively (*P* for trend < 0.001). Subgroup analyses showed a similar pattern. Among DM patients, hyperlipidemia was as a high risk of mortality as non-hyperlipidemia (hazard ratio 1.02, 95%CI 0.73 to 1.43) during the overall follow-up period, but from 48-month follow-up onwards, hyperlipidemia patients had 3.60 (95%CI 1.62 to 8.01)-fold higher risk of all-cause mortality than those non-hyperlipidemia (*P* interaction = 1.000).

**Conclusions:**

PD patients with DM coexisting with hyperlipidemia were at the highest risk of all-cause mortality, followed by DM patients and hyperlipidemia patients, and hyperlipidemia may have an adverse effect on long-term survival in DM patients.

## Introduction

Lipid abnormalities are prevalent in the dialysis population and are influenced by several factors, such as diabetes, renal replacement modalities (hemodialysis and peritoneal dialysis), dietary regimens, and drug use [1, 2]. Hemodialysis can moderately reduce renal hyperlipidemia, and peritoneal dialysis patients show more obvious dyslipidemia than hemodialysis patients, which may be due to the metabolic interference of peritoneal dialysis fluid [1, 3–5].

Diabetes mellitus (DM) is the leading cause of end-stage renal disease in the United States, accounting for approximately 44% of incident cases in dialysis settings [6]. DM patients on dialysis have poor survival (34% over 5 years), worse than those with glomerular disease or hypertension [7]. Compared with dialysis patients without DM, DM patients have a 1.4-fold higher risk of mortality [8]. Hyperlipidemia is prevalent in DM patients on dialysis. Although lipid-modifying medications are associated with a lower risk of cardiovascular events in non-dialysis DM patients [9], the association between hyperlipidemia and mortality is inconsistent in DM patients undergoing hemodialysis [9]. A large prospective study has not shown a benefit of statin therapy in reducing cardiovascular events among DM patients on hemodialysis [10]. However, the authors of this study reported that from a 48-month follow-up onwards, patients with lipid-lowering therapy had a better survival rate than those without lipid-lowering therapy using survival curves. Another study reported that in DM patients undergoing hemodialysis, if the low-density lipoprotein before treatment was more than 145 mg/dl, atorvastatin could significantly reduce the risk of death from any cause [11]. A clear association of DM coexisting with hyperlipidemia and mortality can help stratify the risk of death in peritoneal dialysis patients. However, to date, there is no study on these associations. Therefore, in the present study, associations between DM coexisting with hyperlipidemia and mortality were examined in patients on continuous ambulatory peritoneal dialysis (CAPD).

## Subjects, materials, and methods

### Study design and population

A retrospective cohort study with 3073 incident CAPD patients from five peritoneal dialysis centers in three provinces in China was performed between January 1, 2005, and December 31, 2018. To enhance the generalizability of the findings in the CAPD population, patients younger than 18 years of age or those with less than a 3-month follow-up were excluded. The study was approved by the Human Ethics Committee of each hospital (application ID: [2019]088), consistent with the ethical principles of the Declaration of Helsinki. Written informed consent was obtained from all eligible patients.

### Data collection and definitions

Two trained nurses recorded demographic data, comorbidities, medications, and laboratory parameters at baseline. Two experts evaluated comorbidities by face-to-face interviews and medical records. A total of 2369 (80.6%) patients were on medication, including calcium channel blockers, beta-blockers, angiotensin II receptor blockers/angiotensin-converting enzyme inhibitors (ACEI/ARBs), diuretics, statins, and aspirin. Laboratory parameters included hemoglobin, serum albumin, serum uric acid, estimated glomerular filtration rate (eGFR), cholesterol, triglyceride, high-density lipoprotein, low-density lipoprotein, and high-sensitivity C-reactive protein [hs-CRP]).

DM was defined as (1) 2-h plasma glucose ≥200 mg/dL during an OGTT, (2) HbA1c ≥ 6.5%, (3) fasting plasma glucose ≥126 mg/dL, (4) patients with typical symptoms of hyperglycemia, hyperglycemia crisis, or random blood glucose ≥200 mg/dL, or (5) using glucose-lowering drugs. In the absence of unequivocal hyperglycemia, criteria 1 to 3 should be confirmed by repeated testing [12]. Hyperlipidemia was defined as (1) serum cholesterol levels ≥4.7 mmol/L, (2) triglyceride levels ≥2.3 mmol/L, or (3) low-density lipoprotein levels ≥4.1 mmol/L [13]. Patients who meet one of these three items are defined as having hyperlipidemia. The 169 (8.3%) patients with pre-existing CVE to prevent recurrence of CVD episodes who were receiving lipid-lowering drugs were not considered to have hyperlipidemia. Hypertension was defined as systolic blood pressure > 140 mmHg, diastolic blood pressure > 90 mmHg or the use of antihypertensive medications [14]. General hypertension (primary hypertension) was taken into consideration in the present study. CVD was defined as coronary heart disease, congestive heart failure, arrhythmias, cerebrovascular disease, or peripheral vascular disease [15]. Current smoking was defined as at least one cigarette a day, and current alcohol consumption was defined as > 20 g of ethanol a day [16]. The Chronic Kidney Disease Epidemiology Collaboration equation was used to calculate eGFR [17].

### Outcomes and follow-up

The primary outcome was all-cause mortality. Causes of death were identified by death certificates and medical records. Patients were considered CAPD failures if they died within 3 months from transferring to hemodialysis to death and were not censored.

In light of the new evidence that statin treatment initiation is not recommended in dialysis patients [10, 18, 19], hyperlipidemia patients without lipid-lowering therapy in this study did not receive a new statin treatment when starting CAPD. For clinical purposes (not specifically for this study), all patients needed to return to each center at least quarterly for an overall medical assessment. Eligible patients were followed up until death, CAPD cessation, an 8-year follow-up, or June 30, 2019. Patients who transferred to hemodialysis ≥ 3 months, received renal transplantation, transferred to other dialysis centers, were lost to follow-up, had an 8-year follow-up, or those after June 30, 2019, were considered to be censored.

### Statistical analysis

The incidence rate was calculated by dividing the proportion of events by the total effective observation time in the risk, which is converted to the number of episodes per 1000 years. Data are shown as the mean ± standard deviation (SD), median (interquartile range, IQR) or number (%). Eligible patients were assigned to four groups: group 1 (patients without DM and hyperlipidemia), group 2 (patients with only hyperlipidemia), group 2 (patients with only DM), and group 4 (patients with DM coexisting with hyperlipidemia). Chi-squared, one-way ANOVA, or Kruskal–Wallis tests were used to test for differences in categorical or continuous factor variables among groups. Multiple logistic regression analyses were conducted to examine the adjusted odds ratios of DM coexisting with hyperlipidemia, DM, and hyperlipidemia compared with no comorbidities. The factors in the multinomial logistic regression included demographic data, hypertension, pre-existing CVD, and laboratory parameters.

Kaplan-Meier curves were used to examine the difference in the cumulative mortality hazard among the four groups over the follow up. Four Cox proportional hazards regression models were conducted to examine the association between DM coexisting with hyperlipidemia and mortality: model 1, unadjusted; model 2, model 1 plus demographic and clinical characteristics; model 3, model 2 plus medications; model 4, model 3 plus laboratory variables. A trend across groups and interactions between age, sex, comorbidities, and all-cause mortality was examined. Statistical analyses were conducted with GraphPad Software 8.0 (GraphPad Prism Software Inc., San Diego, California), Stata 15.1. statistical software (StataCorp, College Station, TX), and the R package 3.6.0 (https://www.r-project.org/), and a *P* value < 0.05 was considered significant.

## Results

### Baseline characteristics

Of 3073 potential participants, 42 patients < 18 years of age were excluded, and 92 with a < 3-month follow-up were excluded. The remaining 2939 patients were eligible for this study.

Of the 2939 patients with a median age of 50.0 (IQR 39.0–61.0) years, 1697 (57.7%) were men, 549 (18.7%) had DM, 533 (18.1%) had hyperlipidemia, 1915 (65.2%) had hypertension, and 410 (14.0%) had pre-existing CVD. In this cohort study, 178 patients (6.1%) were assigned to group 4, 371 (12.6%) to group 3, 355 (12.1%) to group 2, and 2035 (69.2%) to group 1. Table 1 shows that compared with group 1, group 4 was more likely to be elderly, have a higher body mass index, systolic BP, hemoglobin, and cholesterol, have lower diastolic BP levels, be female, have hypertension, have pre-existing CVD, and take medications.

**Table 1:** Baseline demographic characteristics, medications, and laboratory parameters

Variables	No comorbidity	Hyperlipidemia	DM	DM plus hyperlipidemia	*P*-value
N	2035	355	371	178	
Age (IQR), years	47.0 (37.0–57.0)	50.0 (39.0–61.0)	60.0 (52.0–67.0)	61.0 (52.2–68.0)	< 0.001
Men, %	1191 (58.5%)	176 (49.6%)	232 (62.5%)	98 (55.1%)	0.003
Body mass index (SD), kg/m^2^	22.2 ± 5.5	23.5 ± 14.9	23.4 ± 3.7	23.9 ± 3.4	< 0.001
Systolic BP (SD), mmHg	148.8 ± 25.8	150.7 ± 24.7	152.9 ± 25.6	157.2 ± 23.2	< 0.001
Diastolic BP (SD), mmHg	88.6 ± 16.2	88.9 ± 14.8	82.1 ± 13.2	82.9 ± 14.4	< 0.001
24-h urine volume (IQR), ml	800.0 (500.0–1200.0)	800.0 (425.0–1200.0)	800.0 (500.0–1200.0)	800.0 (462.5–1287.5)	0.969
Current smoking, (%)	192 (9.4%)	45 (12.7%)	36 (9.7%)	21 (11.8%)	0.239
Current alcohol consumption, (%)	73 (3.6%)	19 (5.4%)	9 (2.4%)	7 (3.9%)	0.209
Hypertension, (%)	1210 (59.5%)	226 (63.7%)	317 (85.4%)	162 (91.0%)	< 0.001
Pre-existing CVD, (%)	169 (8.3%)	64 (18.0%)	105 (28.3%)	72 (40.4%)	< 0.001
Calcium channel blockers, (%)	1446 (71.1%)	299 (84.2%)	291 (78.4%)	165 (92.7%)	< 0.001
Beta blockers, (%)	790 (38.8%)	187 (52.7%)	133 (35.8%)	103 (57.9%)	< 0.001
Diuretics, (%)	111 (5.5%)	8 (2.3%)	70 (18.9%)	11 (6.2%)	< 0.001
ACEI/ARBs, (%)	604 (29.7%)	156 (43.9%)	156 (42.0%)	96 (53.9%)	< 0.001
Aspirin, (%)	109 (5.4%)	19 (5.4%)	74 (19.9%)	42 (23.6%)	< 0.001
Statins, (%)	190 (9.3%)	83 (23.4%)	79 (21.3%)	64 (36.0%)	< 0.001
Hemoglobin (SD), g/dL	9.0 ± 2.8	10.0 ± 2.7	9.5 ± 2.9	10.2 ± 2.5	< 0.001
Serum albumin (SD), g/dL	3.5 ± 0.6	3.4 ± 0.5	3.4 ± 0.6	3.5 ± 0.5	0.619
Serum uric acid (SD), mg/dL	7.0 ± 2.4	6.9 ± 2.2	6.6 ± 2.2	6.9 ± 2.5	0.076
eGFR (IQR), mL/min/1.73 m^2^	6.4 (4.7–8.2)	6.3 (4.7–8.3)	6.7 (4.9–8.9)	6.5 (4.9–8.2)	0.071
Cholesterol (IQR), mmol/L	3.9 (3.0–4.7)	3.8 (3.1.1–4.7)	3.9 (3.1–4.7)	4.1 (3.4–4.9)	0.035
Triglyceride (IQR), mmol/L	1.0 (0.6–1.7)	1.0 (0.6–1.7)	1.1 (0.7–1.8)	1.1 (0.7–1.8)	0.923
High-density lipoprotein (IQR), mmol/L	1.0 (0.8–1.3)	1.0 (0.8–1.3)	1.0 (0.8–1.3)	1.1 (0.9–1.3)	0.826
Low-density lipoprotein (IQR), mmol/L	2.1 (1.3–3.0)	2.1 (1.3–3.0)	2.1 (1.1–3.1)	2.2 (1.3–3.3)	0.316
hs-CRP (IQR), mg/L	4.2 (1.9–13.1)	4.5 (2.0–17.4)	5.0 (2.1–20.8)	4.2 (1.9–13.2)	0.068

The variables represented by the mean ± SD were tested by ANOVA, and the variables represented by IQR were tested by nonparametric tests. *DM* diabetes mellitus, *CVD* cardiovascular disease, *BP* blood pressure, *eGFR* estimated glomerular filtration rate, *hs-CRP* high-sensitivity C-reactive protein, *SD* standard deviation, *IQR* interquartile range

### Factors and DM coexisting with hyperlipidemia, DM, and hyperlipidemia

Table 2 shows that after adjusting for confounding factors, pre-existing CVD, higher body mass index levels, and hemoglobin were independently associated with DM coexisting with hyperlipidemia, DM, and hyperlipidemia using multinomial logistic regression. Additionally, elderly age, hypertension, pre-existing CVD, higher body mass index levels, systolic BP, hemoglobin, and lower levels of diastolic BP were independently associated with a high risk of DM coexisting with hyperlipidemia. Notably, being female was independently associated with hyperlipidemia, and lower levels of serum uric acid were independently associated with DM.

**Table 2:** Associations between variables at baseline and DM coexisting with hyperlipidemia, DM, and hyperlipidemia using the multinomial logistic regression

	No comorbidity	Hyperlipidemia			DM			DM plus hyperlipidemia		
	Reference	Coef.	OR	95% CI	Coef.	OR	95% CI	Coef.	OR	95% CI
Age, per increase 10 years	1.0 (ref.)	0.006	1.06	0.98 to 1.15	0.046	1.58	1.45 to 1.73	0.050	1.64	1.45 to 1.86
Women, men as a reference	1.0 (ref.)	0.530	1.70	1.33 to 2.17	−0.234	0.79	0.61 to 1.02	0.160	1.17	0.83 to 1.66
Body mass index, per increase 1 kg/m2	1.0 (ref.)	0.026	1.03	1.01 to 1.04	0.026	1.03	1.01 to 1.04	0.031	1.03	1.01 to 1.05
Systolic BP, per increase 10 mmHg	1.0 (ref.)	0.002	1.02	0.97 to 1.07	0.012	1.13	1.07 to 1.19	0.018	1.20	1.12 to 1.29
Diastolic BP, per increase 10 mmHg	1.0 (ref.)	0.002	1.02	0.93 to 1.11	−0.031	0.73	0.67 to 0.80	−0.030	0.74	0.65 to 0.84
Hypertension, yes/no	1.0 (ref.)	0.500	1.05	0.82 to 1.35	1.116	3.05	2.21 to 4.20	1.503	4.49	2.63 to 7.69
Pre-existing CVD, yea/no	1.0 (ref.)	0.779	2.18	1.57 to 3.03	0.775	2.17	1.60 to 2.94	1.240	3.46	2.39 to 5.01
Hemoglobin, per increase 1 g/dL	1.0 (ref.)	0.110	1.12	1.08 to 1.16	0.047	1.05	1.01 to 1.09	0.122	1.13	1.07 to 1.19
Serum uric acid, per increase 1 mg/dL	1.0 (ref.)	−0.004	1.00	0.95 to 1.04	− 0.068	0.93	0.89 to 0.98	−0.014	0.99	0.92 to 1.06

The following variables at baseline were included in the multinomial logistic regression model: age, sex, body mass index, systolic BP, diastolic BP, current smoking, current alcohol consumption, 24-h urine volume, hypertension, pre-existing CVD, hemoglobin, serum albumin, serum uric acid, eGFR, cholesterol, triglyceride, high density lipoprotein, low density lipoprotein, and hs-CRP

*DM* diabetes mellitus, *CVD* cardiovascular disease, *BP* blood pressure, *eGFR* estimated glomerular filtration rate, *hs-CRP* high-sensitivity C-reactive protein, *OR* odds ratio, *CI* confidence interval

### Observational period and mortality

The overall follow-up period was 10,122.2 patient-years, with a median of 35.1 (IQR 17.9–61.7) months. At the end of the study, 519 patients (17.7%) had died, including 258 (8.8%) CVD deaths, 54 (1.8%) infection deaths, 9 (0.3%) gastrointestinal bleeding deaths, 16 (0.5%) tumor deaths, 93 (3.2%) other death causes, and 82 (2.8%) unknown death causes. Additionally, 353 patients (12.0%) transferred to hemodialysis, 153 (5.2%) received renal transplants, 26 (0.9%) transferred to other centers, and 100 (3.4%) were lost to follow-up. The incidence rates of all-cause mortality were 142.3, 118.2, 39.5, and 36.5/1000 patient-years among groups 4, 3, 2, and 1, respectively.

### DM coexisting with hyperlipidemia, DM, and hyperlipidemia and mortality

**Fig. 1:**
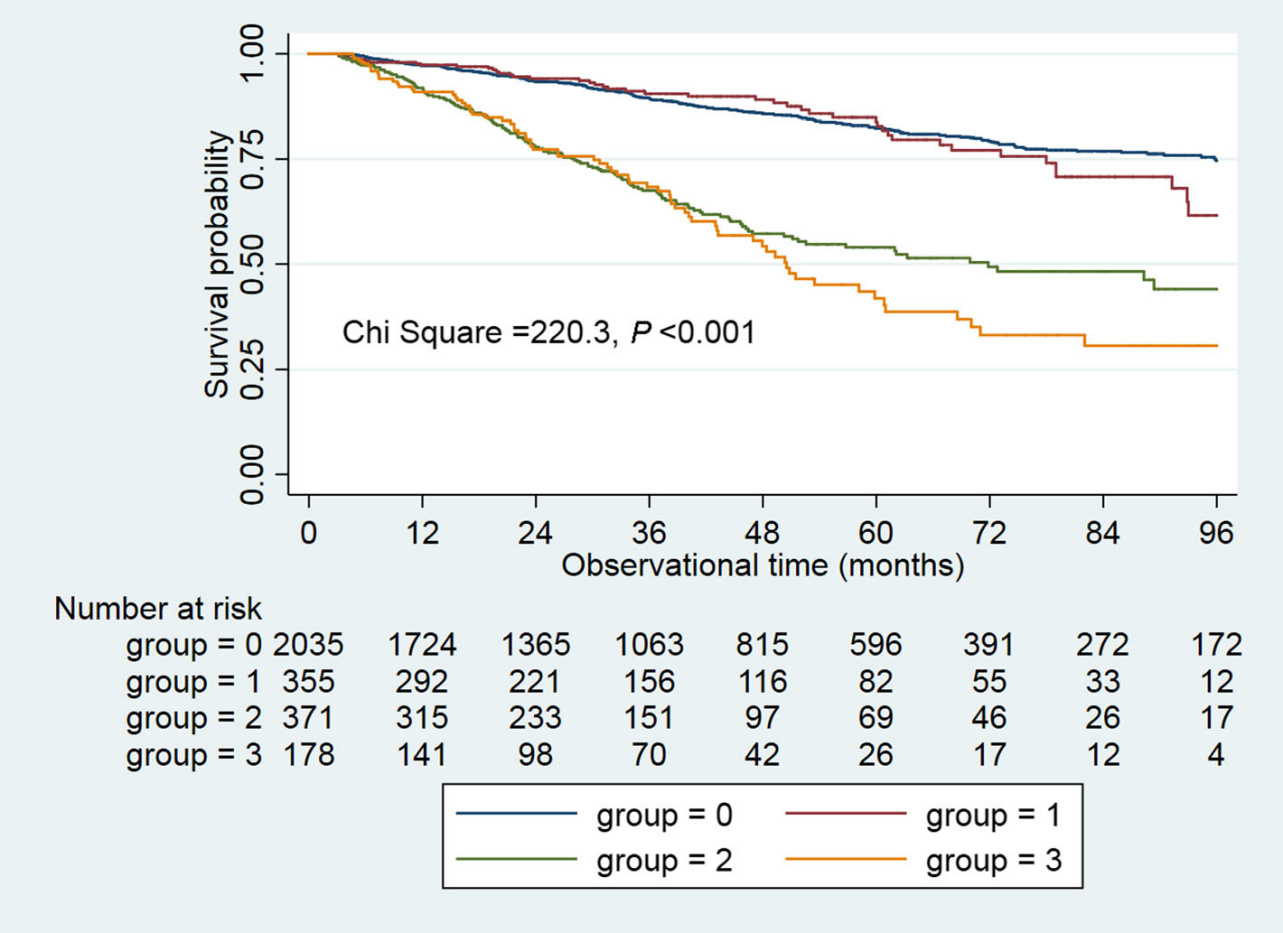
Cumulative survival curves among four groups. Survival curves showed that from 48-month follow up onwards, DM plus hyperlipidemia group had poor survival than DM group, suggesting among DM patients, hyperlipidemia had a adverse effect on long-term survival. DM, diabetes mellitus

Survival analysis found that cumulative survival differed significantly among the four groups (Chi square = 220.3, *P* < 0.001; Fig. 1). Among DM patients from a 48-month follow-up onwards, hyperlipidemia patients had poorer cumulative survival than non-hyperlipidemia patients, suggesting that hyperlipidemia had an adverse effect on the long-term prognosis of DM patients.

**Fig. 2:**
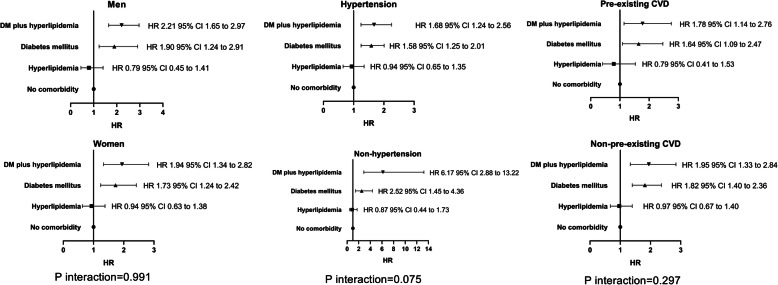
Associations among DM coexisting with hyperlipidemia, DM, and hyperlipidemia and all-cause mortality among subgroups. Covariables in the Cox proportional hazards regression model were included age, sex, body mass index, systolic BP, diastolic BP, current smoking, current alcohol consumption, 24-h urine volume, hypertension, pre-existing CVD, medications, hemoglobin, serum albumin, serum uric acid, eGFR, cholesterol, triglyceride, high-density lipoprotein, low-density lipoprotein, and hs-CRP, except the subgroup variable. DM, diabetes mellitus; CVD, cardiovascular disease; BP, blood pressure; eGFR, estimated glomerular filtration rate; hs-CRP, high-sensitivity C-reactive protein; HR, hazards ratio; CI, confidence interval

Table 3 shows that compared to group 1, groups 4, 3, and 2 had a 1.93- (95% CI 1.45 to 2.56), 1.86- (95% CI 1.45 to 2.32), and 0.90- (95% CI 0.66 to 1.24) times higher risk of all-cause mortality, respectively, after adjusting for factors in model 4 (*P* trend < 0.001). The subgroups of men, women, hypertension, non-hypertension, pre-existing CVD, and non-pre-existing CVD showed similar patterns (Fig. 2). There was no interaction among DM coexisting with hyperlipidemia, DM, and hyperlipidemia on all-cause mortality (*P* = 0.570). *P* values for the interactions were > 0.05 for subgroups by sex (*P* = 0.991), hypertension (*P* = 0.075), and pre-existing CVD (*P* = 0.297), suggesting that the increased risk of all-cause mortality associated with DM coexisting with hyperlipidemia, DM, and hyperlipidemia was evident regardless of these factors.

**Table 3:** Adjusted HRs for all-cause mortality among different Cox proportional hazards regression models

	Model 1				Model 2				Model 3					Model 4		
	Coef.	SE	HR	95%CI	Coef.	SE	HR	95%CI	Coef.	SE	HR	95%CI	Coef.	SE	HR	95%CI
No comorbidity			1.0 (ref.)													
Hyperlipidemia	0.092	0.177	1.09	0.80 to 1.50	−0.103	0.147	0.90	0.66 to 1.24	−0.101	0.151	0.90	0.66 to 1.24	−0.103	0.155	0.90	0.66 to 1.24
DM	1.191	0.349	3.29	2.67 to 4.05	0.608	0.208	1.84	1.47 to 2.29	0.607	0.209	1.84	1.47 to 2.29	0.621	0.217	1.86	1.49 to 2.32
DM plus hyperlipidemia	1.389	0.534	4.01	3.09 to 5.21	0.667	0.282	1.94	1.46 to 2.57	0.662	0.300	1.94	1.46 to 2.57	0.656	0.313	1.93	1.45 to 2.56
*P* for trend			< 0.001				< 0.001				< 0.001				< 0.001	

Model 1, unadjusted; model 2, model 1 plus age, sex, body mass index, systolic BP, diastolic BP, current smoking, current alcohol consumption, 24-h urine volume, hypertension, and pre-existing CVD; model 3, model 2 plus medications; model 4, model 3 plus hemoglobin, serum albumin, serum uric acid, eGFR, cholesterol, triglyceride, high-density lipoprotein, low-density lipoprotein, and hs-CRP

*DM* diabetes mellitus, *CVD* cardiovascular disease, *BP* blood pressure, *eGFR* estimated glomerular filtration rate, *hs-CRP* high-sensitivity C-reactive protein, *SE* standard error, *HR* hazard ratio, *CI* confidence interval

Table 4 shows that compared with hyperlipidemia patients, DM patients had a 2.37-fold (95% CI 1.66 to 3.39) higher risk of all-cause mortality in model 4 (*P* interaction = 1.000). Among DM patients, patients with hyperlipidemia had a similar risk of all-cause mortality compared to those without hyperlipidemia (HR = 1.02, 95% CI 0.73 to 1.43) during the overall follow-up period, but from the 48-month follow-up onwards, patients with hyperlipidemia had a significantly higher risk of all-cause mortality than those without hyperlipidemia (HR = 9.62, 95% CI 5.06 to 18.29) after adjusting for confounding factors (*P* interaction = 1.000), which was consistent with the finding of survival curves. Among hyperlipidemia patients, DM patients had a higher risk of all-cause mortality than non-DM patients (HR 2.61, 95% CI 1.69 to 4.04). In addition, among non-DM patients, hyperlipidemia patients had a similar risk of all-cause mortality (HR 0.97, 95% CI 0.70 to 1.35) as non-hyperlipidemia patients.

**Table 4:** Association among DM and hyperlipidemia and all-cause mortality^*^

	Model 1				Model 2				Model 3				Model 4			
	Coef.	SE	HR	95%CI	Coef.	SE	HR	95%CI	Coef.	SE	HR	95%CI	Coef.	SE	HR	95%CI
Hyperlipidemia group			1.0 (ref.)													
DM group	1.090	0.319	3.01	2.14 to 4.21	0.811	0.238	2.24	1.58 to 3.18	0.811	0.238	2.24	1.58 to 3.18	0.858	0.251	2.37	1.66 to 3.39
*Among DM patients*																
The follow-up period																
Hyperlipidemia (yes/no)	0.434	0.127	1.20	0.91 to 1.61	0.391	0.115	1.08	0.80 to 1.45	0.391	0.121	1.14	0.84 to 1.54	0.369	0.108	1.02	0.73 to 1.43
From 48-moth follow up onwards																
Hyperlipidemia (yes/no)	1.046	0.306	2.89	1.37 to 6.07	1.046	0.306	2.89	1.34 to 6.22	1.303	0.382	3.60	1.62 to 8.01	1.303	0.382	3.60	1.62 to 8.01
*Among non-DM patients*																
Hyperlipidemia (yes/no)	0.366	0.107	1.01	0.82 to 1.51	−0.103	0.095	0.90	0.65 to 1.24	−0.101	0.097	0.91	0.65 to 1.27	−0.170	0.103	0.97	0.70 to 1.35
*Among hyperlipidemia patients*																
DM (yes/no)	1.368	0.401	3.78	2.60 to 5.50	0.948	0.278	2.62	1.74 to 3.96	1.024	0.300	2.83	1.86 to 4.30	0.945	0.277	2.61	1.69 to 4.04

^*^P interaction = 1.000

Model 1, unadjusted; model 2, model 1 plus age, sex, body mass index, systolic BP, diastolic BP, current smoking, current alcohol consumption, 24-h urine volume, hypertension, and pre-existing CVD; model 3, model 2 plus medications; model 4, model 3 plus hemoglobin, serum albumin, serum uric acid, eGFR, cholesterol, triglyceride, high-density lipoprotein, low-density lipoprotein, and hs-CRP

*DM* diabetes mellitus, *CVD* cardiovascular disease, *BP* blood pressure, *eGFR* estimated glomerular filtration rate, *hs-CRP* high-sensitivity C-reactive protein, *SE* standard error, *HR* hazard ratio, *CI* confidence interval

## Discussion

In this study, among CAPD patients, baseline DM coexisting with hyperlipidemia was more robustly associated with all-cause mortality than either DM or hyperlipidemia alone. This association was significant regardless of sex, hypertension, and pre-existing CVD. Interestingly, during the overall follow-up period, among DM patients, hyperlipidemia patients were at a similarly high risk of all-cause mortality as non-hyperlipidemia patients. However, from the 48-month follow-up onwards, hyperlipidemia patients had a 3.60-fold higher risk of all-cause mortality than non-hyperlipidemia patients, suggesting that hyperlipidemia had an adverse effect on the long-term survival of DM patients. Among non-DM patients, hyperlipidemia did not affect mortality irrespective of the follow-up duration. In addition, DM patients had a 2.37-fold higher risk of all-cause mortality than hyperlipidemia patients.

Studies in the general population have shown that reducing total cholesterol and low-density lipoprotein levels can significantly reduce cardiovascular events and mortality [20–22]. However, in dialysis patients, several epidemiological investigations and randomized clinical trials have failed to show the beneficial effect of lipid-lowering therapy in reducing CVD, although low-density cholesterol levels are significantly reduced [10, 18, 19, 23]. Dyslipidemia is commonly observed in dialysis patients and is characterized by abnormal composition and concentrations of plasma lipoproteins [24]. People are paying increasing attention to the harmful effects of glucose-based PD solutions on various metabolic changes (such as lipid abnormalities) [25]. In fact, several studies have shown that compared with hemodialysis patients, the lipid distribution of PD patients has stronger atherosclerosis [26–29]. The serum total cholesterol and low-density cholesterol levels of PD patients are usually elevated, while the serum levels of cholesterol and low-density cholesterol levels in hemodialysis patients are normal or low. In addition, PD patients’ serum triglyceride levels are much higher than those of hemodialysis patients, and the serum high-density cholesterol levels of both patient populations are usually low [26–29]. Elevated non-high-density lipoprotein cholesterol can predict atherosclerotic cardiovascular events in hemodialysis patients [30].

This was an observational cohort study from a nationwide dialysis registry in Japan of 45,390 hemodialysis patients without pre-existing CVD with 44,190 person-years. Higher cholesterol and high-density cholesterol levels in hemodialysis patients were associated with a lower risk of all-cause mortality. A previous study examined the relationship between time-varying serum lipid concentration and all-cause and CVD mortality in a 10-year follow-up of 749 PD patients. In contrast to the general population, in PD patients, lower cholesterol and low-density cholesterol levels over time were significantly associated with adverse events and CVD mortality. Although lower triglyceride and high-density lipoprotein cholesterol concentrations were associated with a significantly higher risk of all-cause mortality, they failed to show any significant association with CVD mortality [31]. In the present study, we defined hyperlipidemia according to lipid management in the Chinese population. Among CAPD patients, DM coexisting with hyperlipidemia at baseline was more strongly associated with all-cause mortality than DM or hyperlipidemia alone. Notably, hyperlipidemia harmed long-term survival in DM patients but had no effect in non-DM patients. In addition, DM was independently associated with a higher risk of all-cause mortality in hyperlipidemia patients. In this study, the diagnosis of hyperlipidemia was based on the 2016 Chinese Guideline for the Management of Hyperlipidemia in Adults, which only included higher lipid profiles. Thus, patients with lower lipid profiles may be assigned to the non-hyperlipidemia group; lower lipid profiles are prevalent and associated with a higher risk of mortality in dialysis [32–34]. This study’s assignment may affect the association among DM coexisting with hyperlipidemia, DM, and hyperlipidemia and mortality. Additionally, in this study, there was no significant difference in lipid profiles between patients with hyperlipidemia and those without, with the exception of cholesterol. This may be because a patient with high cholesterol may have low triglycerides or low-density lipoprotein. In the future, clinical trials with PD patients should consider hyperlipidemia as a primary or secondary endpoint.

The results of subgroup analyses highlighted the consistency in the association between DM coexisting with hyperlipidemia and the risk of all-cause mortality across men and women, hypertension and non-hypertension, and pre-existing CVD and non-pre-existing CVD. However, it is noteworthy that the strength of the association between DM coexisting with hyperlipidemia and the risk of all-cause mortality may vary by sex, hypertension, and pre-existing CVD. Interestingly, among DM patients, patients with hyperlipidemia had a similar risk of mortality as those without hyperlipidemia during the overall follow-up period. However, among DM patients, from the 48-month follow-up onwards, patients with hyperlipidemia had a 3.60-fold higher risk of mortality than those without hyperlipidemia. Statins accounted for 36.0% of patients with DM coexisting with hyperlipidemia and 9.3% in their counterparts. The lower percentiles of statin use were due to the fact that statin treatment initiation is no longer recommended in dialysis patients [10, 18, 19].

### Study strengths and limitations

The study’s strengths were its sizeable multicenter sample size, the generalized inclusion and exclusion criteria of the sample, and rigorous multivariate regression analyses.

This study had some limitations. First, this was a retrospective cohort study involving potential unexplained confounding factors and selection bias. Even after adjusting for confounding variables at baseline, we did not reach a conclusion about the potential causality between comorbidities and mortality. Second, defining hyperlipidemia was challenging. In this study, given the effect of ethnicity and district characteristics on the Chinese CAPD population, the diagnosis of hyperlipidemia was based on the 2016 Chinese Guideline for Managing Hyperlipidemia in Adults [13], which only included higher lipid profiles. The definition may underestimate the prevalence of dyslipidemia. Third, because dynamic changes in serum lipids were not recorded, the effect of the serum lipid profile on mortality was not examined. Nonetheless, if patients had significant serum lipid disorders during the follow-up period, an integrated approach including lifestyle modifications, nutritional supplements, anti-inflammatory drugs, and improved dialysis therapy to improve serum lipid disorders was conducted [35]. Finally, to strengthen the generalizability of the findings in the CAPD population, patients younger than 18 years of age or patients with a follow-up period of less than 3 months were excluded. Furthermore, all eligible patients were from China, suggesting that the findings may lack generalizability to other ethnic population settings.

## Conclusion

This study showed that among CAPD patients, patients with DM coexisting with hyperlipidemia at the start of CAPD were at the highest risk of mortality, followed by DM patients and hyperlipidemia patients, and DM patients had a higher risk of mortality than hyperlipidemia patients. In addition, among hyperlipidemia patients, DM was independently associated with a high risk of mortality. It is worth noting that hyperlipidemia may harm long-term survival in DM patients but has no effect in non-DM patients. The findings suggest that clear associations between DM coexisting with hyperlipidemia, DM, and hyperlipidemia and mortality may further help with the stratification of the risk of mortality in CAPD patients with comorbidities.

## Data Availability

The datasets used and/or analyzed during the current study are available from the corresponding author on reasonable request.

## Abbreviations

CAPD: continuous ambulatory peritoneal dialysis
DM: diabetes mellitus
CVD: cardiovascular disease
BP: blood pressure
eGFR: estimated glomerular filtration rate
hs-CRP: high-sensitivity C-reactive protein
SD: standard deviation
IQR: interquartile range
OR: odds ratio
HR: hazard ratio
CI: confidence interval
